# Predicting Physical Exercise Adherence in Fitness Apps Using a Deep Learning Approach

**DOI:** 10.3390/ijerph182010769

**Published:** 2021-10-14

**Authors:** Oscar Jossa-Bastidas, Sofia Zahia, Andrea Fuente-Vidal, Néstor Sánchez Férez, Oriol Roda Noguera, Joel Montane, Begonya Garcia-Zapirain

**Affiliations:** 1 eVIDA Research Group, University of Deusto, 48007 Bilbao, Spain; sofia.zahia95@deusto.es (S.Z.); mbgarciazapi@deusto.es (B.G.-Z.); 2 Department of Physical Activity and Sport Sciences, FPCEE Blanquerna, Ramon Llull University, 08022 Barcelona, Spain; cienciayfisioterapia@gmail.com (A.F.-V.); joelmm@blanquerna.url.edu (J.M.); 3 Mammoth Hunters S.L., 08036 Barcelona, Spain; nestor@mhunters.com (N.S.F.); oriol@mhunters.com (O.R.N.); 4 Blanquerna School of Health Sciences, Ramon Llull University, 08025 Barcelona, Spain

**Keywords:** deep learning, regression, adherence, mHealth, eHealth, fitness app, physical activity

## Abstract

The use of mobile fitness apps has been on the rise for the last decade and especially during the worldwide SARS-CoV-2 pandemic, which led to the closure of gyms and to reduced outdoor mobility. Fitness apps constitute a promising means for promoting more active lifestyles, although their attrition rates are remarkable and adherence to their training plans remains a challenge for developers. The aim of this project was to design an automatic classification of users into adherent and non-adherent, based on their training behavior in the first three months of app usage, for which purpose we proposed an ensemble of regression models to predict their behaviour (adherence) in the fourth month. The study was conducted using data from a total of 246 Mammoth Hunters Fitness app users. Firstly, pre-processing and clustering steps were taken in order to prepare the data and to categorize users into similar groups, taking into account the first 90 days of workout sessions. Then, an ensemble approach for regression models was used to predict user training behaviour during the fourth month, which were trained with users belonging to the same cluster. This was used to reach a conclusion regarding their adherence status, via an approach that combined affinity propagation (AP) clustering algorithm, followed by the long short-term memory (LSTM), rendering the best results (87% accuracy and 85% F1_score). This study illustrates the suggested the capacity of the system to anticipate future adherence or non-adherence, potentially opening the door to fitness app creators to pursue advanced measures aimed at reducing app attrition.

## 1. Introduction

Physical inactivity and sedentary behaviour have been described as a worldwide pandemic [1]. More than a quarter of the world’s adult population is considered to be physically inactive [2] and consequently at 20–30% increased risk of death, compared to people who are sufficiently active [2]. Inactivity has been associated with at least 35 different chronic conditions [3] and is considered a leading cause of mortality in non-communicable diseases [1,2], with approximately 3.2 million deaths per year [4]. Parallel to health-related concerns, this situation also has a direct economic global cost which was estimated at $54 billion per year, in the year 2013 [4], with an additional $14 billion attributable to lost productivity [5].

Current WHO guidelines recommend that adults undertake 150–300 min of moderate intensity activity per week which, in fact, is met by as little as 25% of the total world adult population [4]. Physical activity should additionally be complemented by regular muscle strengthening, a recommendation met by only 17.3% of adults in Europe [6], and by a reduction in sedentary behaviour [7]. Generally speaking, it is women and people living in high-income countries who account for the worst share [2]. Recent data from Germany has shown that ∼80% of adults fail to meet total activity and strengthening guidelines [6], which would seem to be in line with the aforementioned statements. The healthcare industry seems to now have realised the extent of the problem, which is proven by the fact that prescribing physical activity is what is most often done by healthcare workers [8]. But in spite of this, and since 2001, adherence keeps declining by as much as 5% (years 2001–2016) in high-income countries [2]. In order to bring about a shift in this trend, the WHO has made it a worldwide priority to reduce physical inactivity rates by 15% by the year 2030 [4].

Some authors have pointed to high attrition rates as a key element in explaining increased sedentary behaviour [9]. Given the widespread use of mobile technology, apps constitute highly feasible means for delivering health interventions and may offer an opportunity to reach that part of the population that is predisposed to starting an exercise programme [10]. Several studies, including some systematic reviews, have attempted to analyse adherence to exercise apps. Nonetheless, in the early stages of our research [11,12], we noticed a remarkable lack of consensus regarding the conceptualisation of this term. Most commonly, adherence has been assessed in clinical research environments involving individuals with health conditions that followed some type of therapeutic intervention. To this extent, an expected “dosage” of exercise is typically prescribed, and adherence is expressed in terms of frequency, with 80–99% of the recommended exercise dosage being considered “satisfactory” or “adherent” [13]. “Yang et al.” determined that the definition of adherence to physical activity apps could be categorized into 4 dimensions: (i) frequency of app usage; (ii) intention/motivation to sustain use of the app; (iii) degree of function use within the app; and (iv) duration of app usage [11]. Previous researchers had, however, used the word “engagement” to refer to duration of usage, number of challenges/programmes started and number of physical activity log days [14]. Earlier in time, a meta-analysis by Cugelman et al. concluded that the term adherence was the opposite to attrition and therefore equalled the percentage of people who continue to use the app over time [15]. They further called this “intervention adherence”. In contrast, they defined “study adherence” as the proportion of participants in a study at a given time, compared to baseline. Already back in 2011, authors agreed that coding “intervention adherence” was more challenging as they used different criteria to measure it [15]. Adherence has also been defined as the number of weeks before the participant becomes inactive for an entire week [16], while other authors have chosen to call this “retention” [17].

Our preliminary literature search revealed studies on adherence to PA guidelines, but not specifically on user behaviour in fitness apps. Some of the studies retrieved included biological age prediction, sports performance forecasting and human physical activity recognition [18,19,20,21]. El-Kassabi et al. [19] proposed different DL models to forecast an athlete’s performance in sports tournaments and to guide their strategies accordingly to obtain improved results. They were able to evaluate the effectiveness of the athlete’s training by predicting their race time results upon completing each additional training. Their results showed that the DL model managed to improve race time prediction accuracy over the baseline ML model, such as in the case of linear regression. In [21], the authors proposed a model to predict physical activity based on an LSTM, with the aim of providing personalized services based on data collected from mobile devices. To provide personal services and to select the model variables, the characteristics and surrounding data circumstances were considered, and the following were some of the variables they took into account in order to provide such customized services: sleep hours, travel distances, mean heart rate, cadence, weather, temperature, mean daily temperature, humidity. The LSTM was trained to learn the dynamic characteristics and to then predict users’ physical activity, while the performance model was evaluated using the root mean square error (RMSE). The model obtained good mean RSME results. Similarly, researchers in [20] used LSTM to process raw data from gyroscope and accelerometer sensors, and to classify six activities involved in daily living. In [18], a deep convolutional LSTM approach was used to estimate biological age in human adults.

Big data techniques could be valuable for the analysis of user behavioural features and could lead to behavioural change encouraging actions. Deep learning (DL) has recently outperformed other machine learning (ML) methods on many fronts, with image recognition, audio classification and natural language processing being just a few of the many examples [22,23,24,25,26]. For its part, Modern DL provides a powerful, adaptive and flexible framework for supervised learning and can be considered as the way to automate predictive analytics. By adding more components (layers and units within a layer) to the network, DL is able to work with more complex problems [27]. Provided there is a large number of training samples with their respective labels and sufficiently large and well-designed model architecture, many of the tasks that were traditionally performed by individuals (e.g., mapping input to output vectors) can now be developed through DL [27]. Each algorithm hierarchically applies a non-linear transformation to its input and uses what it learns to create an output, and then iterations continue until the output has reached an acceptable level of accuracy. Once training has been completed (i.e., back propagation has been repeatedly applied to update the weights in the model, so as to achieve the desired accuracy with the training data), the resulting model can then be used to make predictions with new data that the model has not been previously exposed to. This all means that the DL approach will require much less feature engineering and be less reliant on domain knowledge.

The major purpose of our study may be described as follows: “It is possible to predict training adherence behaviour for a subsample of MH app users, over a given period of time, by processing data from previous training sessions with artificial intelligence algorithms.” User behaviour was analysed in terms of training behaviour, whereby a prediction was established for the fourth month by taking into consideration user activity over the first three months. Section 2 explains the materials and methods used to conduct the project, Section 3 describes the experimental setup, along with the results obtained, and Section 4 provides a discussion of those results in terms of positive and negative highlights, as well as future directions. Lastly, Section 5 offers the conclusions drawn from this pilot study.

## 2. Materials and Methods

### 2.1. Data Acquisition

This was an observational, retrospective pilot study. The research protocol observed the principles set out in the Declaration of Helsinki and study approval was granted by the Research Ethics Committee at the Ramon Llull University (nr.1920003P). 777 participants who voluntarily agreed to participate and granted their informed consent were recruited from the company Mammoth Hunters S.L.

**Figure 1:**
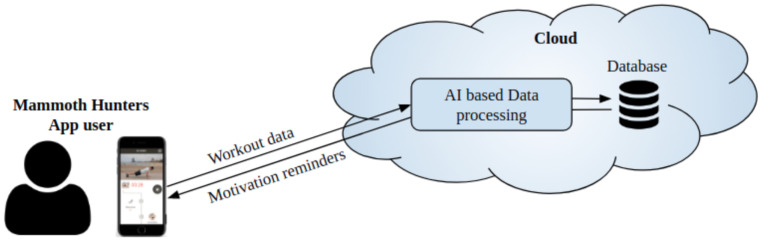
Illustration of MH app usage.

MH is a smartphone application that provides physical workouts. Upon registration, users are presented with a series of workouts that can form either part of a predefined training programme or personalized according to an individual’s characteristics and preferences. Users do their workouts by following instructional videos on their screens, with indications regarding performance of exercise and number of repetitions or time taken per exercise. Upon completion of each workout, a summary of total repetitions and total workout time is displayed on the screen. MH stores all training sessions in its databases, as seen in Figure 1. It additionally sends information to an analytic platform called MixPanel, where additional information (e.g., IP, operating system, phone device) is stored. MH obtained explicit consent from all participants in this research project and ensured all registries remained confidential by sharing only anonymized data for analysis. The description of the data acquired from the MH app is shown in Table 1.

523 individuals in total met the inclusion criteria (i.e., MH app users older than 18 years old). These users were almost equally split between males and females, with an average age of 40 years, an average weight of 71kg, average body mass index (BMI) around 25 and average body fat of 24%. Almost 85% of the participants were equally split between a desire to lose weight and increase muscle mass, as shown in the following Table 2.

Based on previous literature [28,29,30,31,32] a period of four months was established as a determining factor and only those participants who had been subscribed for at least four consecutive months after enrolment (n = 243) were ultimately considered eligible for the prediction analyses.

Table 3 shows the description of the per-month data for the 246 users. From this analysis, it can be seen that the average duration of the training sessions is between 300 and 400 s per day/month. However, it is not possible to conclude anything with regard to the mean, since standard deviation clearly indicates that the data is very dispersed. This fact is ratified by the difference between the maximum and minimum values observed for each month. Therefore, in order to have an idea of data distribution, we decided to obtain the frequency of the data in time intervals, as described in Table 4.

Table 4 shows the frequency of user numbers in ranges of exercise time per day, over the four months. For example, column one shows that 41 users did not exercise on a daily basis in the first month. It also reflects that the majority of users trained between the ranges of 0 to 300 and 300 to 1800 s in the first month. In other words, most individuals trained less than 30 min, and very few trained more than half an hour. It is worth stressing that, on average, most users tended to do less exercise in the fourth month.

### 2.2. Proposed Framework

**Figure 2:**
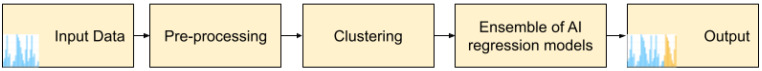
Proposed framework to determine adherence to training in the MH fitness app.

**Figure 3:**
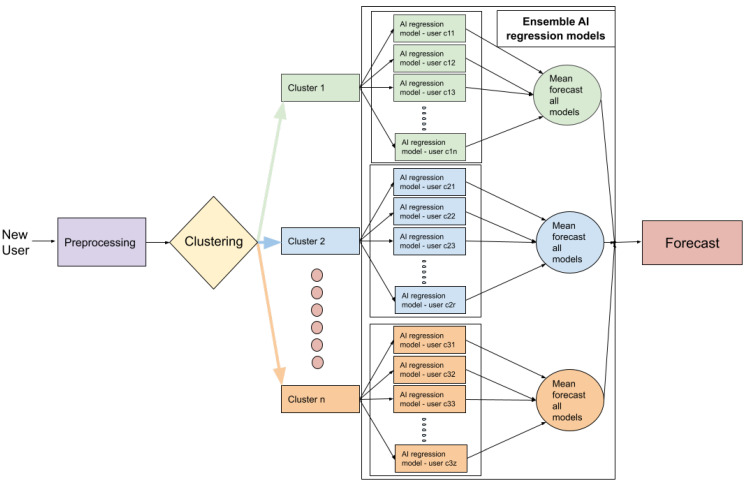
Proposed architecture to determine user adherence to physical exercise in the MH fitness app.

The goal of this study was to develop a model that was able to predict user adherence (continuation of training sessions) to the MH fitness app, at month four. Figure 2 shows a general diagram of our proposed framework, which is further explained in Figure 3.

Table 5 and Table 6 below show the pseudocode of the algorithm that was used in this study, while Table 5 shows the proposed framework for the training process, which can be summarised by the creation of the regression ensemble models of the users who underwent similar training in the first three months. Table 6 shows the proposed framework for testing new users. This process can be summarised in terms of the determination of adherence or non-adherence in the case of new users who sign up for the MH fitness app. In order to achieve this, every new user is categorized into a cluster, and the mean of the ensemble comprising people that similarly exercised during their first three months is set as the output. Table 5 further explains this process.

#### 2.2.1. Input Data

The raw data received from the MH app contained user information on age, gender, city, weight, height, body fat, body type, body fat target, individual’s goal, profile creation, programmes, current programme and sessions, as shown in Table 1. To meet our study goals, we needed historical data, so as to be able to design an intelligent system that could learn from past workout behaviour and would then be able to forecast future ones. We selected the data about sessions, given that it provided us with time-dependent information from the time the user performed their first training session. The original data contained in this field corresponded to a JSON file and contained several workout details.

#### 2.2.2. Pre-Processing

In data analytics, and in the case of intelligent systems, it is very important to identify any incorrect or corrupted data, as this could significantly affect the decision-making process. There is already plenty of evidence from fields such as finance, business, health systems and smart cities to suggest that incorrect or inaccurate decisions may have unfortunate consequences [33]. In order to identify corrupted or duplicate data and to build a reliable dataset, several techniques (e.g., data cleaning) could be used. In this paper, a pre-processing stage was first developed, in order to create new variables that allowed us to represent user evolution over time (i.e., determining the length of a workout session from start to finish). Upon completion of the pre-processing stage, a new dataset was built with the duration of training sessions, and so this variable contained the accumulated workout time, in seconds. It should be noted that the information corresponded to the duration of the actual workout session, as opposed to the time spent on the app.

Given the differences between users in app registry duration (some users had been active for two years, others for six months, etc.), the next step required was to determine a longitudinal time period. In previous literature, we found that other authors had used periods of 12 or 16 weeks for their PA-related research [28,29,30,31,32] and so, based on their findings, we decided to focus our system on a period of 16 weeks. From these, the first 12 weeks were used to train the system and the last four weeks were used to predict the workout sessions. A summary of the data can be seen in Table 7.

#### 2.2.3. Clustering

The clustering block represents an approach which was chosen in order to categorize users into groups, based on their training frequency during the first three months. Instead of using all the models trained with the entire group of user’ sessions, the clustering approach grouped models trained with data from users with similar exercise features.

Three clustering approaches were used: K-means, Balanced Iterative Reducing and Clustering using Hierarchies (BIRCH), and Affinity Propagation (AP).

- K-means: K-means is one of the preferred methods in unsupervised ML strategies. It is widely used in manufacturing, education and business [34], and is based on minimization of the sum of distances between each object and the centroid of its group or cluster. Once the number of clusters (K) is chosen, the first initialisation step is to establish K centroids among the data. Then, samples are assigned to their closest centroids. Next, the positions of the centroids are updated, so that the distances between the elements of each cluster may be minimized. The assignment and update process is then repeated until no points change clusters, or equivalently, until the centroids stay the same. In order to assign a point to the closest centroid, a proximity measure that quantifies the notion of closest is required. Commonly, it is the Euclidean distance that is used for this, and so the goal is to find the objective function which minimizes the squared distance of each point to its closest centroid [35]. The sum of squared errors (SSE) calculates the error of each point (i.e., Euclidean distance from each point to the closest centroid) [35,36]. The SSE is defined by the Equation (1):
  (1) $$SSE=\sum_{i=1}^{k}\sum_{x\in C_{i}}\mathrm{dist}{\left(m_{i},x\right)}^{2}$$
  where dist is the standard Euclidean distance, *x* is an object, $C_{i}$ is the $i^{t}h$ cluster and $m_{i}$ is the centroid (mean) for cluster $C_{i}$ what minimizes the SSE of the cluster is the mean, defined by the Equation (2):
  (2) $$m_{i}=\frac{1}{n}\sum_{x\in c_{i}}x$$
  where *n* corresponds to the number of objects in the $i^{t}h$ cluster. The Basic K-means functionality is described in Table 8.
- BIRCH: This is a non-supervised algorithm. Due to its ability to find good clustering with only a single data scan, it is especially suitable for larger datasets or streaming data [37]. This characteristic was especially relevant to our research, since we expect to obtain a larger dataset in the near future, and we were in need of a process that would facilitate the upscaling of our application.
  In order to understand how the BIRCH algorithm works, the concept of cluster feature (CF) needs to be introduced. CF is a set of three summary statistics which represent a single cluster, from a set of data points. The first statistic, count, quantifies how many data values are present in the cluster. The second, linear sum, is a measurement which represents cluster location. Finally, squared sum refers to the sum of the squared coordinates that represents the spread of the clusters. The last two statistics are equivalents to mean and variance of the data point [37]. BIRCH is frequently explained in two steps: (1) building the CF tree, and (2) global clustering.
  **Phase 1–Building the CF Tree:** Firstly, the data is loaded into the memory by building a CF Tree, for which purpose a sequential clustering approach is used. Thus, the algorithm simultaneously scans and records the data, and then determines whether a point should be added to an existing cluster, or a new cluster should be created.
  **Phase 2–Global clustering:** Secondly, an existing clustering algorithm is applied to the sub-clusters (the CCF lead nodes), so as to assemble these sub-clusters into clusters. This could, for instance, be achieved using the agglomerative hierarchical method.
  The basic BIRCH algorithm is described in Table 9.
- Affinity propagation: this clustering method was proposed by Fred and Dueck in 2007, and works with the similarity matrix. Points that appear close to each other have high similarity while those that are furthest have low similarity [38]. Unlike others, the AP method is not required as a parameter, although it is commonly used in experiments where many clusters are needed. AP works with three matrices: similarity matrix, responsibility matrix and availability matrix.
  **Similarity matrix:** this is the first matrix obtained, and is calculated by negating the sum of the squares of the difference between participants [39]. Thus, the elements in the main diagonal of the similarity matrix equal 0 (zero) and a value needs to be selected in order to fill these. Consequently, the algorithm will converge around a few clusters if the selected value is low, and vice-versa, insofar as the algorithm will converge with many clusters, in the case of high selected values.
  **Responsibility matrix:** once the similarity matrix has been calculated, the next step is to calculate the responsibility matrix, given by the Equation (3).
  (3) $$r\left(i,k\right)\leftarrow s\left(i,k\right)-\max_{k\prime \;\;\;\mathrm{such}\;\mathrm{that}\;k^{\prime }\neq k}\left\{a\left(i,k^{\prime }\right)+s\left(i,k^{\prime }\right)\right\}$$
  where *i* corresponds to the number of rows and *k* to the number of columns in the associated matrix.
  **Availability matrix:** the availability matrix is then calculated. All elements are set to zero, and Equations (4) and (5) are then used to calculate elements off the diagonal.
  (4) $$a\left(k,k\right)\leftarrow \sum_{i\prime \;\mathrm{such}\;\mathrm{that}\;i^{\prime }\neq k}\max\left\{0,r\left(i^{\prime },k\right)\right\}$$
  (5) $$a\left(i,k\right)\leftarrow \min\left\{0,r\left(k,k\right)+\sum_{i\;\mathrm{such}\;\mathrm{that}\;i^{\prime }\in \left[i,k\right\}}\max\left\{0,r\left(i^{\prime },k\right)\right\}\right\}$$
  In essence, the Equation (4) corresponds to the sum of all values in the columns that are above 0, except for values which are identical for both rows and the given column.
  **Criterion matrix:** Finally, the algorithm calculates the criterion matrix. This equals the sum of the availability matrix and the responsibility matrix at that location, and is given by (6).
  (6) c(i,k)←r(i,k)+a(i,k)
  The highest criterion value of each row is designated as the exemplar. The pseudocode of AP can be seen in Table 10.

#### 2.2.4. Regression Models

**Figure 4:**
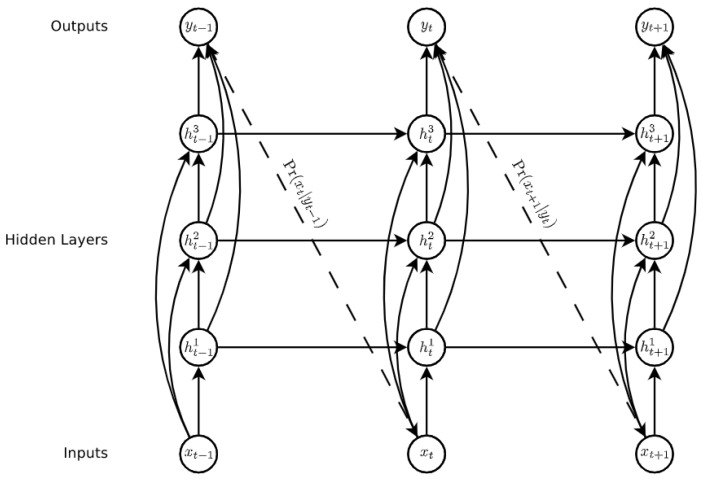
Deep recurrent neural network prediction [42].

**Figure 5:**
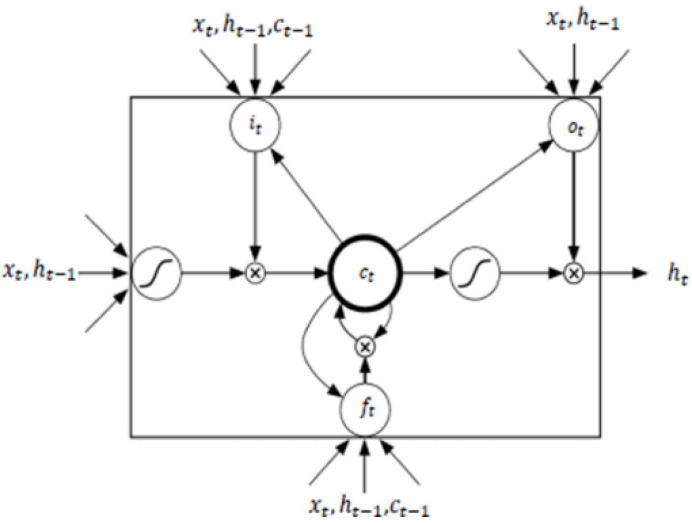
LSTM CELL based on [42].

- Recurrent Neural Networks and Long-Short-Term Memory model: Recurrent Neural Networks (RNN) are a family of neural networks used to process sequential data [27], which are well-known and widely used to process time series data and natural language processing [40,41]. These networks are built upon the idea of using the output of the previous neuron in the network along with the next input of the sequence as input to the next. This ability gives the network the opportunity to model sequences. It facilitates modelling cases in which the relationships between variables are not simply parallel, but rather sequential (the value of a given variable at one time may determine the value of another at a later or earlier time). Sequential data can be trained as: complete sequences, forward or backward sequences, or a set of them.
  Figure 4 illustrates the basic architecture of a recurrent neural network. Given an input vector sequence $x=x_{1},x_{2},....,x_{t}$, passed through to bunch of N recurrently connected hidden layers. The first hidden vector sequences are calculated as $h^{n}=\left(h_{1}^{n},\ldots ,h_{T}^{n}\right)$ and the output vector sequence $y=\left(y_{1},\ldots ,y_{T}\right)$. Where N = 1, the architecture is simply reduced to a single layer. Hidden layer connections are calculated as follows:
  (7) $$h_{t}^{1}=H\left(W_{ih^{1}}x_{t}+W_{h^{1}h^{1}}h_{t-1}^{1}+b_{h}^{1}\right)$$
  (8) $$h_{t}^{n}=H\left(W_{ih^{n}}x_{t}+W_{h^{n-1}h^{n}}h_{t}^{n-1}+W_{h^{n}h^{n}}h_{t-1}^{n}+b_{h}^{n}\right)$$
  where *H* corresponds to the hidden layer functions, *W* equals the weight matrices (e.g., $W_{h^{1}h^{1}}$ is the recurrent connection at the first hidden layer), *b* denotes bias vectors (e.g., $b_{y}$ is the output bias vector). Then the output is computed by:
  (9) $${\hat{y}}_{t}=b_{y}+\sum_{n=1}^{N}W_{h^{n}y}h_{t}^{n}$$
  LSTM is one of the most famous types of RNN architecture [43]. It can memorize for long and short periods of time using a gating mechanism which makes it possible to control the information that has to be kept over time, the duration it has to be kept for and the time that it can be read through the memory cell [44]. The architecture of an LSTM cell, as described in [42], is shown in Figure 5, where H is implemented by the following composite function:
  (10) $$i_{t}=\sigma \left(W_{xi}x_{t}+W_{hi}h_{t-1}+W_{ci}c_{t-1}+b_{i}\right)$$
  (11) $$f_{t}=\sigma \left(W_{xf}x_{t}+W_{hf}h_{t-1}+W_{cf}c_{t-1}+b_{f}\right)$$
  (12) $$o_{t}=\sigma \left(W_{xo}x_{t}+W_{ho}h_{t-1}+W_{co}c_{t}+b_{o}\right)$$
  (13) $$c_{t}=f_{t}c_{t-1}+i_{t}\tanh\left(W_{xc}x_{t}+W_{hc}h_{t-1}+b_{c}\right)$$
  (14) $$o_{t}=\sigma \left(W_{xo}x_{t}+W_{ho}h_{t-1}+W_{co}c_{t}+b_{o}\right)$$
  (15) $$h_{t}=o_{t}\tanh\left(c_{t}\right)$$
  where σ is the logistic function **$i_{t}$, $f_{t}$**, **$o_{t}$**, **$c_{t}$**, **$h_{t}$** correspond to the input gate, forget gate, output gate, memory cell and hidden state at time *t* respectively, and **$x_{t}$** refers to the input of the system at time *t*.
- Support vector machine (SVM): this is an algorithm based on statistical learning and which has gained great popularity over the last decade. It is useful in several classification and regression problems [36,45,46]. SVM takes the structural risk minimization principle into account and attempts to find the locations of decision boundaries (also known as hyperplanes), which produce optimal separation among the classes [47,48].
  This paper used a support vector regression (SVR), which refers to a generalization of classification problems, where the model returns continuous values. SVM generalization to SVR is achieved by introducing an ϵ-insensitive region around the function, referred to as the ϵ-tube. The tube then reformulates the optimization problem in order to find a tube value which best fits the function, while balancing model complexity and prediction error [49]. SVR problem formulation derives from a geometrical representation, and its continuous-value functions could be approximately represented by:
  (16) $$y=f\left(x\right)=\langle w,x\rangle +b=\sum_{i=1}^{M}w_{i}x_{i}+b,y,b\in R,x,w\in R^{M}$$
  (17) $$f\left(x\right)={\left[\begin{array}{c} w\\ b \end{array}\right]}^{T}\left[\begin{array}{c} x\\ 1 \end{array}\right]=w^{T}x+b\;x,w\in R^{M+1}$$
  where *w* and *b* correspond to the weight and bias vectors, respectively. In spite of this, and in real applications, data tends to be non-linear and separable, and Kernel functions are therefore used to extend the concept of the optimal hyperplane.
  In multidimensional data, x augments by one, and b is included in the w vector for a simple mathematical notation (see Equation (17)). The multivariate regression in SVR then formulates the function approximation problem as an optimization which attempts to find the narrowest tube centred around the surface [49]. The objective function is shown below in Equation (18), where w equals the magnitude of the normal vector to the surface.
  (18) $$\min_{w}\frac{1}{2}{∥w∥}^{2}$$
  The Grid search method was used to tune the hyperparameters, whereby three different kernel functions (i.e., radial basis function, polynomial kernel and sigmoid kernel) were used. These three kernel methods are defined by the Equations (19)–(21), respectively.
  (19) $$\begin{array}{ccc} \; & K\left(x_{i},y_{i}\right)= & \exp\left(-\gamma {∥x_{i}-y_{i}∥}^{2}\right). \end{array}$$
  (20) $$\begin{array}{ccc} \; & K\left(x_{i},y_{i}\right)= & {\left(\gamma \left(x_{i},y_{i}\right)+r\right)}^{d} \end{array}$$
  (21) $$\begin{array}{ccc} \; & K\left(x_{i},y_{i}\right)= & \left(\gamma \left(x_{i},y_{i}\right)+r\right) \end{array}$$
  where γ is the influence on classification outcomes; large values for γ leads to over-fitting and small values result in under-fitting [50].

#### 2.2.5. Ensemble Models

Once we had trained the LSTM and SVM algorithms and used the grid search for each user to tune the hyperparameters, we then tried to develop a strategy that allowed us to use the trained ML models and combine several base models in order to produce one optimal predictive model. We decided to use an ensemble approach for this purpose. An ensemble consists of a set of individually trained classifiers or regressors whose predictions are combined in order to obtain better results than with single methods [51]. In this research, we used an approach similar to that used for bagging methods [52] and, as such, our objective was to compensate for the error generated by some models while at the same time combining the models into clusters based on the similarity in terms of their training data. The final prediction was the mean output value of combined models from the same cluster, as shown in Figure 3.

#### 2.2.6. Output

**Figure 6:**
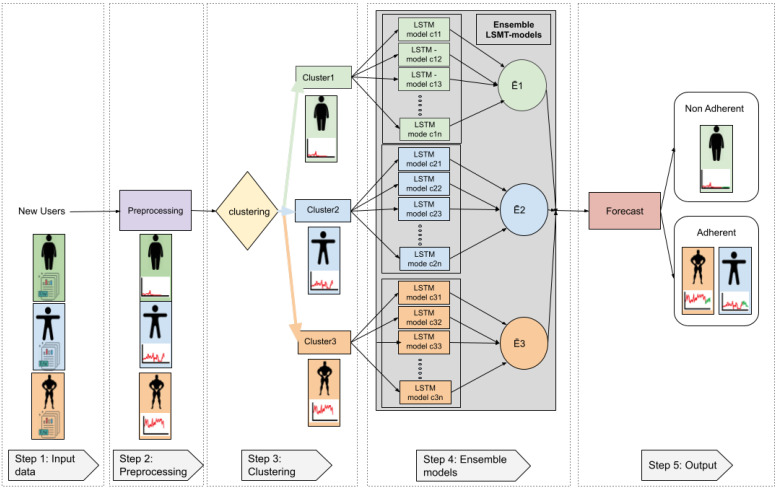
Example of the proposed system with three different user categories.

For a better understanding of the output of the proposed framework, we shall refer to Figure 6, which contains an example that simulates three different users: users with high exercise frequency, users with medium exercise frequency, and users with low exercise frequency. The full steps a user has to go through in the system are explained below.

- Step 1 (Input data): Raw data from the three different users is given as input to the system. Users can belong to one of the three aforementioned categories (depicted in different colours).
- Step 2 (Pre-processing): In the pre-possessing step, all data cleaning procedures, as well as other operations, are applied in order to obtain the data from workout sessions.
- Step 3 (Clustering): When using the K-means algorithm, if three clusters are selected and the characteristics are the mean of accumulated seconds per day over three months, clusters categorize the users into three groups: people with high PA (orange colour), people with medium PA (blue colour), and people with low PA (green colour).
- Step 4 (Ensemble models): Assuming we are using the LSTM as the regression method, data corresponding to the first three months of use is given to the corresponding LSTM ensembles (orange, blue or green). The ensembles then use pre-trained models to calculate all regressions and the output will be the mean of the corresponding ensemble; Ē1: mean ensemble 1 (green), Ē2: mean ensemble 2 (blue), Ē3: mean ensemble 3 (orange).
- The system output corresponds to the average regression of the models within a given cluster. Since our aim was to determine adherence to training using a fitness app, we turned again to literature in order to follow a rule that defined user adherence. Previous researchers have established that exercise-derived health benefits taper off after 4–5 weeks of training cessation [53,54,55,56,57]. Taking this into account, we determined that a user would be considered non-adherent if he/she showed no training activity over a full month (the fourth month).

## 3. Experiments and Results

This section shows all the implementation and results obtained throughout all stages of the project.

### 3.1. Implementation

We used Phyton programming language, version 3.8.5. For code writing, we used the Jupiter client-server application. All the dependencies were installed using the Conda package manager, in its version 4.9.2. Additionally, we used the open source sklearn library [58] for clustering algorithms, while DL algorithms were implemented using the Keras library, version 2.4.3.

### 3.2. Clustering

Three different algorithms were used to allocate new users into similar groups, while taking some of their characteristics (data training as calculated during the first 90 days) into account. Their features and corresponding descriptions are shown in Table 11.

The purpose of creating these variables was to provide the clustering algorithms with information so that, whenever a new user signs up, our framework extracts these features based on the first three months, and the clustering algorithm will categorize the user based on their training behaviour in the app. The variables mean_week_n were calculated from week 1 until week 12, and Table 11 shows a simplification of this process.

**Figure 7:**
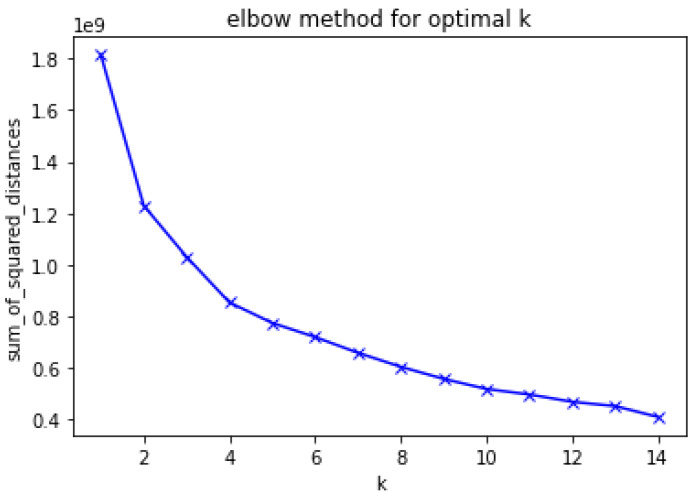
Elbow curve for optimal k.

Three methods were selected, to test different scenarios for clustering algorithms. We first wanted to test the scenario where few clusters were expected, and chose the K-mean algorithm for such purpose, as no more than 3 or 4 groups were expected. The elbow curve in Figure 7 confirms that 3, 4 or 5 clusters are sufficient. The BIRCH algorithm caught our attention, due to its ability to work with larger samples, which could prove useful for our future work. Finally, we used the AP in scenarios where several groups of people did exercise. With our data, clusters were automatically calculated, and results showed variations between 15 and 30 groups.

#### 3.2.1. K-Means

The parameter to adjust in the K-means clustering algorithm was the number of clusters, and we used the elbow method to set up the number of clusters, as shown in Figure 7.

From the curve above, we can note that the optimal number of clusters is k = 4. However, we also tested the results for k = 3. The curve in Figure 7 was obtained using three features: (missed_first_month, missed_second_month, missed_third_month).

**Figure 8:**
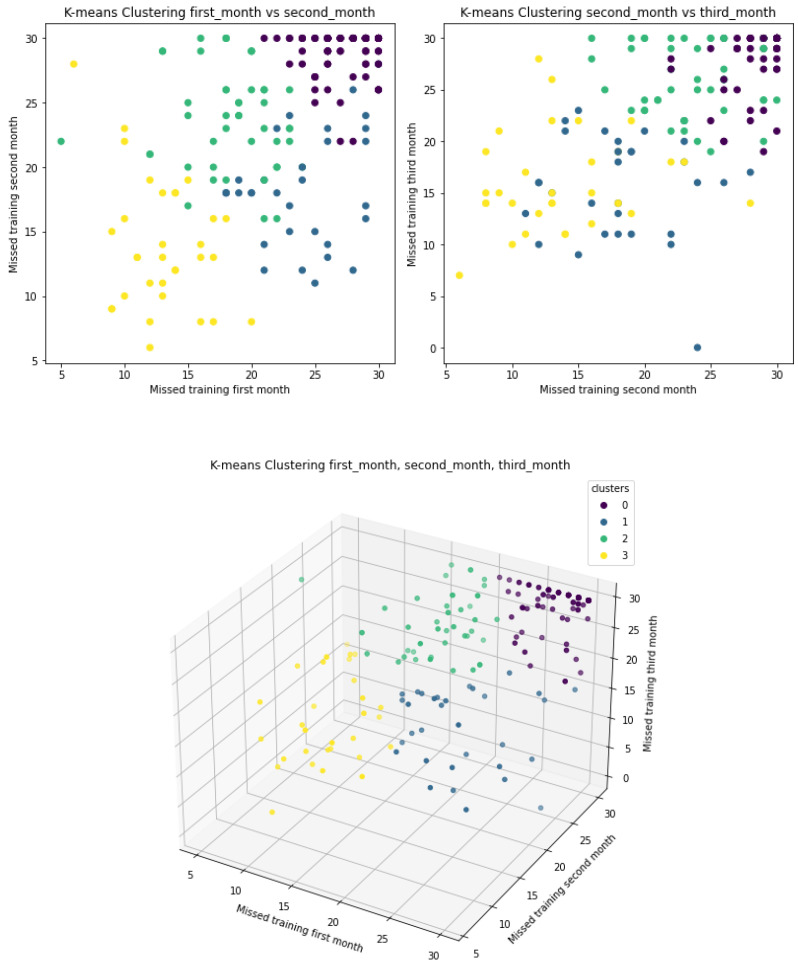
4-means clustering taking “missed first month”, “missed second month” and “missed third month”as features.

As for Figure 8, the top-left scatter plot represents the number of skipped sessions in the first month vs. the number of skipped sessions in the second month. The top-right scatter plot represents the number of skipped sessions in the second month vs. the number of skipped sessions in the third month, while the bottom 3D scatter plot combines the three axes into a single plot. The clusters show that users could be categorized into four groups: 1. users who, for the last three months, have been exercising for an average of one session every two days (yellow colour); 2. users who exercised moderately during the first month, but reduced their PA in the following months (green colour); 3. users with low PA in the first month, but who increased their PA in the second and third months (blue colour); and finally, those who evidenced very low PA across all three months (purple colour). The 3D scatter plot shows the separability between the four clusters.

#### 3.2.2. BIRCH

The parameter used to configure the BIRCH algorithm was the threshold which represents the radius of the subcluster obtained by merging a new sample and the closest subcluster. The radius should be lower than the threshold [58]. Thus, it is important to adjust the threshold because the values can be represented by explicit coordinates in a Euclidean space. Upon building the cluster, special attention should be paid to units. In this dataset, features that depend on the accumulated time (e.g., mean_first_month, mean_week_1...) are expressed in seconds, while those features which depend on the accumulated number of missed workout sessions (e.g., missed_first_month, missed_second_month) do not have units.

**Figure 9:**
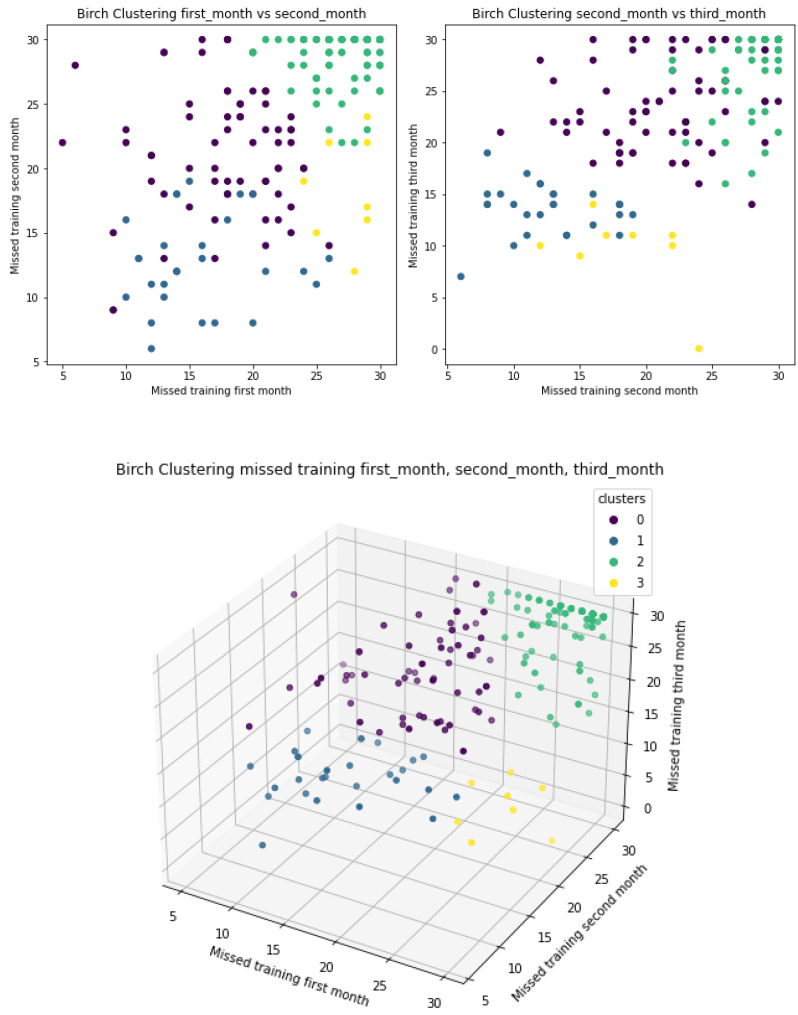
BIRCH clustering taking “missed first month”, “missed second month” and “missed third month” as features.

Figure 9 shows the three scatter plots represent clustering by the BIRCH algorithm. Similarities to clusters by K-means can be identified.

#### 3.2.3. Affinity Propagation

The implementation of the sklearn library for the AP algorithm has two characteristics. On the one hand, when the fit function does not converge, the cluster centres meet in an empty array and then all training samples are labelled as −1. The predict function will classify each label as −1 accordingly. On the other hand, when all training has equal similarities and equal preferences, assignments of cluster centers and labels will depend on preferences. Thus, if the preference is smaller than the similarities, then the fit function will result in a single cluster and label 0 for each cluster. Otherwise, every training sample becomes its own cluster centre and is assigned a unique label [58]. Taking all of the above into account, we tested the AP by changing the damping parameter in the library, it is specified that this value must be in the range between [0.5, 1). With this in mind and following several data tests, we obtained the best results to 0.7 and 0.8 for the damping parameter.

### 3.3. Regression Results

The regression model was one of the most important stages in our research, and the LSTM model was selected for its ability to work with sequences and to memorize long and short term dependencies [43,59]. Here, we were interested in characterizing the training of MH users, with the aim of predicting future workout sessions during the fourth month, based on their training during the first three months.

**Figure 10:**
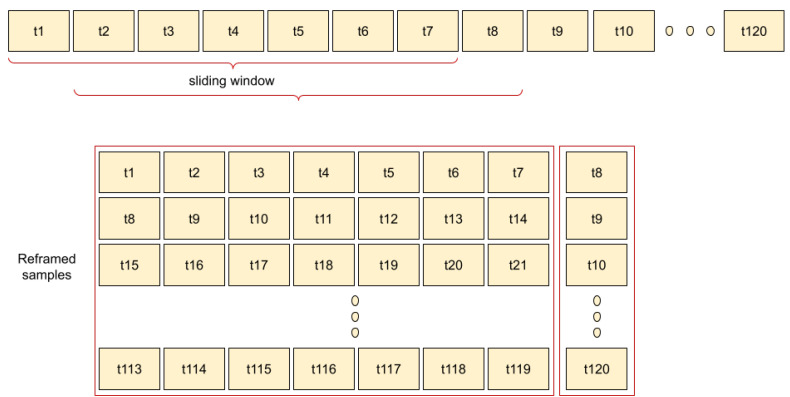
Time series conversion into supervised learning data, for a lookback value of 7.

The first step in this module was to convert time series data into supervised learning data. The conversion procedure used can be seen in Figure 10. A lookback value of 7 was used within the conversion procedure, implying that the LSTM system will learn in 7-day periods, the system output consequently being the eighth day. This process was carried out using the function TimeseriesGenerator from the Keras library.

**Figure 11:**
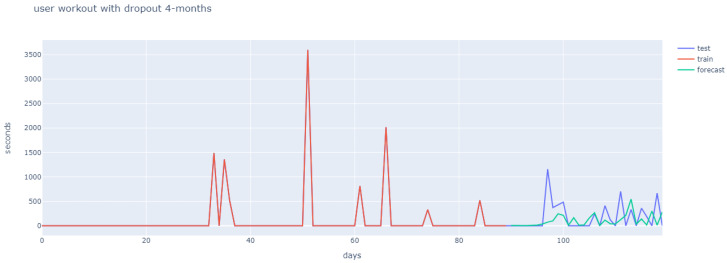
User 1 workout behaviour (four months).

**Figure 12:**
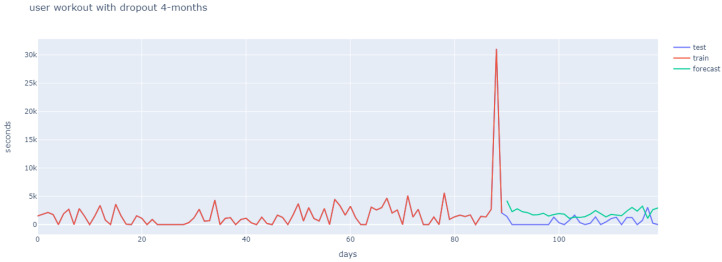
User 2 workout behaviour (four months).

**Figure 13:**
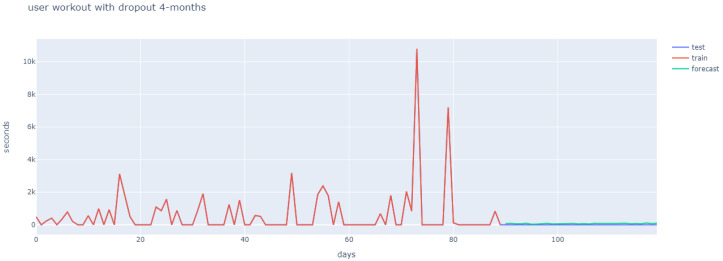
User 3 workout behaviour (four months).

User behaviour differs greatly, and this can be observed in the first 90 days (marked in red), in Figure 11, Figure 12 and Figure 13. Hence, the strategy was to build a general architecture for different types of user, whereby the creation of ensembles with similar users was combined with the previously mentioned clustering stage and the hyperparameter tuning for each user.

Figure 11, Figure 12 and Figure 13 reflect how different user behaviour was found for months 1–3 (in red), and for month 4 (in purple). The fact that different user behaviour was found makes it unnecessary to train and adjust hyperparameters for each newly-arrived user, which justifies the general approach proposed. Additionally, Figure 11 shows the long-short term memory, which is able to ignore the first month of workout sessions and learns from months two and three, so as to obtain the prediction of an adherent user for the fourth month. The LSTM prediction of an adherent user in Figure 12 proved similar, with a small upward shift with regard to test data. Finally, non-adherent user prediction is shown in Figure 13.

#### Hyperparameter Tuning

Given the sophisticated, automatic connections between their inputs and their outputs, neural networks have the ability to learn very complicated patterns [27]. Adding a hyperparameter tuning process to this optimizes the extraction of network parameters and leads to better regression results. There are different techniques used for hyperparameter tuning. Some are based on optimization methods that aim to obtain the best configuration of the networks, including Bayesian optimization to adaptively select configurations [60,61], Hill Climbing- Random Restart and Tabu List algorithms originally [46,62,63]. Other classical methods like grid search have shown great effectiveness. However, they have high computational complexity, especially with a wide range of parameter values that need to be tuned. In this regard, other researchers have explored alternative techniques, such as the suboptimal grid search [64]. In this paper, we used the grid search technique for hyperparameter tuning for LSTM and SVR, since it was necessary to optimize a large number of models with different behaviour.

- LSTM—grid search: following a large number of tests with different users, a wide range of hyperparameters was chosen for which the models generally adjusted the regression curve better. The same hyperparameters and number of neurons were then used for all users. Specifically, three dropout values after the first and second dense layers were applied. Similarly, three batch sizes of values 1,2,4 were used, taking into consideration the number of days in a week. Finally, five neuron values were applied to the first layer (LSTM layer). The range of aforementioned values are highlighted in Table 12.
  Remaining hyperparameters were selected from existing literature (previous work on prediction, even if aimed at different types of application), with high performance in their proposed architectures. Hence, in accordance with the previous explanation, we pursued a hyperparameter tuning strategy in the relevant literature [22,65], with a detailed explanation of all the values in Table 12. The lookback is a parameter which was selected and agreed with the MH team, as it was considered more appropriate for the purpose of analysing the evolution of training over the weeks, as people generally change their routines or lose their motivation within a period of one week [66]. Additionally, following some experiments, we also verified that curves were fitting better with a value of 7 days. The number of epochs was then selected to be 50 after observing in experiments that overfitting was occurring after 50 epochs. Next, based on [67], we selected the number of hidden layers to be 4 after performing several experiments. The activation function selected was Relu, since it resolved the problem of negative values, and had performed well in previous research [68]. The Relu activation function was applied to all layers (including LSTM and dense), except the final one, while early stopping with patience of 15 epochs, was configured in our architecture, in order to avoid over-fitting.
- SVR-grid search: The hyperparameters modified in the case of SVR were kernel, with the choices ‘poly’, ‘rbf’, and ’sigmoid’. Similarly, for the hyperparameter c, a range of 0 to 500 was chosen since the MAE error was not reduced beyond this number with any combination of kernel or other hyperparameters setup. Finally, the hyperparameter gamma, which assigns the scale option, and epsilon, which has a value of 10, were left unchanged.

### 3.4. Classification Results

#### 3.4.1. Validation Metrics

We used the following performance metrics to evaluate the performance of our ensemble approach, precision, specificity and F1_score.

- Confusion matrix: this displays and compares actual values with predicted values of the model. In the context of ML, a confusion matrix is used as a metric to analyse how a machine or deep learning classifier performs on a dataset. It consists of the following 4 elements:

- True Positives (TP): Users who were correctly predicted to exercise in the fourth month.
- True Negatives (TN): Users who were correctly predicted to not exercise in the fourth month.
- False Positives (FP): Users who were predicted to exercise but actually didn’t exercise in the fourth month.
- False Negatives (FN): Users who were predicted to not exercise in the fourth month, but who actually did.

- Accuracy: this is a metric that evaluates the performance of classification models. It indicates the fractions that the model gets right in classifying of correct predictions of adherent and non-adherent users in the total set evaluated. It is defined by (22).

(22) $$\mathrm{Accuracy}=\frac{\mathrm{TP}+\mathrm{TN}}{\mathrm{TP}+\mathrm{TN}+\mathrm{FP}+\mathrm{FN}}$$

- Precision: this is a value which tracks the performance of a model in terms of positive example classification (taking into account the users that were correctly and incorrectly predicted to exercise in the case of the positive class).

(23) $$\mathrm{Precision}=\frac{\mathrm{TP}}{\mathrm{TP}+\mathrm{FP}}$$

- Recall: this equals the number of genuine positive examples (i.e., users who were correctly predicted to exercise), divided by the number of false negatives (i.e., users who were incorrectly predicted to not exercise) and the total number of positive examples (users who were correctly predicted to exercise).

(24) $$\mathrm{Recall}=\frac{\mathrm{TP}}{\mathrm{TP}+\mathrm{FN}}$$

- F1_score: this is a weighted average of recall and precision.

(25) $$F1\_\mathrm{score}=\frac{2\;*\;\mathrm{Precision}\;*\;\mathrm{Recall}}{\mathrm{Precision}+\mathrm{Recall}}$$

- Specificity: this quantifies the TN rate or the number of users that the model defined as not exercising in the fourth month and who, in fact, did not train.

(26) $$\mathrm{Specificity}=\frac{\mathrm{TN}}{\mathrm{TN}+\mathrm{FP}}$$

#### 3.4.2. Results

After all the pre-processing stage, the total number of users in our model equaled 246 (Figure 7). We trained with all 246 users in order to get the most out of the ensembles proposed of which 112 were adherent and 134 were non-adherent, as shown in Table 7. It should be noted that the testing data was never shown to our LSTM models (e.g., last 30 days or fourth month of training). We then tested the model over the last 30 days for each user, using the regression results obtained from all the remaining 245 users.

Table 13 presents the classification results using the regressions with a single ensemble, ignoring the clustering stage. The ensembles output corresponds to the regressions average in the fourth month for all trained models, while the system output (Class 0: Non-adherent and class 1: Adherent) was obtained using the rule mentioned in Section 2.2.6. Table 13 shows high metrics for accuracy and specificity, while metrics for recall and f1_score were not so high. This indicated that the system was unable to correctly classify users who actually exercised during the fourth month, and justified the need to employ a clustering stage, to make it possible to categorize users at the system input. Thus, our results are presented using two regression methods: SVR and LSTM, each one preceded by three different clustering methods.

The following tables contain the results obtained by making combinations with 4 types of characteristics and altering some clustering algorithm parameters. The words in bold reflect the results with higher scores for each combination of parameters and features.

Table 14 shows how the best results were obtained by using the number of skipped sessions trait in the first, second and third month. Additionally, it can be noticed that the recall and f1_score improved, although values remained unacceptable.

The best results were obtained for k = 4 and threshold 0.1. See Table 15. The features which produced the best results were the same as those used in the k-means algorithm. It is also worth noting that the threshold values shown in the table were those which, after running a number of tests, produced the greatest changes. Low threshold values did not create changes in results, although large increases caused results to worsen.

In the AP algorithm, the Damping parameter was modified between the allowed range of 0.5 to 1, and Table 16 clearly reflects how the algorithm did not converge in the case of for values lower than 0.66. For this reason, only values higher than 0.66 were presented. No major changes were found in terms of results.

The following tables show the results obtained using LSTM architecture while changing the clustering method. We can observe that the LSTM model in combination with k-means clustering, obtained significant results when compared to the results using SVR, as seen in Table 17, with a recall value of 70% (acceptable results). Modifying the number of clusters led to no variations in results. However, accuracy did increase by 7%, compared to the best results obtained using SVR and clustering combinations.

In Table 18, the best results for the BIRCH algorithm and LSTM were obtained by taking a combination of the following features: mean of completed sessions in the first, second and third month (in seconds), means of completed sessions in weeks 8–12, and skipped workout sessions, in seconds.

The results of the LSTM model preceded by the AP clustering method are shown in Table 19. It shows that it is possible to obtain good results with several features, such as the mean of completed sessions (seconds) in weeks of training, or skipped workout sessions per month, with accuracy values of up to 86%, and 87%, respectively. Additionally, the best results were obtained using all the combined features, with a Damping value of 0.68.

Finally, the confusion matrix of LSTM model preceded by the AP clustering method which represents the best model is shown in Table 20.

## 4. Discussion

Our results show that our initial purpose, stating that user adherence can be predicted, was correct. In order to achieve this, we used ML to categorize users into groups, and regressions according to DL ensembles, so as to predict user adherence to fitness app training.

Another finding was that the LSTM model outperforms the SVR model (12% accuracy increase), when the former is combined with clustering by Affinity Propagation. This is due to LSTM ability to learn from a series of past observations in order to predict the next sequential value. Adding a clustering block before the regression model has also increased (>15%) accuracy and recall (35%), which means that grouping users into similar categories can help to achieve better predictions of physical activity behaviour in new users.

Both in the case of LSTM and SVR models, the features which better helped to determine whether a user would be adherent to app training or not were mainly the combination of all features extracted, as shown in Table 11. We also obtained good results when clustering by the K-means method while using solely the skipped workout sessions feature. This could intuitively lead one to think that the number of missed workouts could have a greater significance than other features when determining adherence to training, and the latter could be relevant when predicting training behaviour in general. Somewhat in line with our results, previous research has associated mood improvement with the completion of the session, rather than with its duration [69]. If sticking to the training plan leads to improvements in mood, perceived pleasantness and usefulness, the former could be positively affected. And these two factors have been hypothesized to correlate directly with adherence to the training plan [70].

On a different note, the results in confusion matrix showed that the system we propose leads to high numbers of FN predictions. These FN become obvious in those cases where we know that the user completed workouts in month four, while our framework had predicted that they would not. While it may seem like a meaningless mistake, given that the client is in fact not at risk of dropping out, such incorrect predictions could have consequences. For instance, receiving a non-adherence prediction could lead to a series of unnecessary and inadequate motivational strategies, and the consequences of these otherwise unnecessary actions are unknown.

Our results (see Table 19) for FP values showed 5 false positive predictions—i.e., the system predicted that 5 individuals would continue to train during month four, while they actually failed to do so. These predictions showed 95% precision and 96% specificity, which we interpret as being satisfactory. Despite this being a low figure, it still implies that 5 clients could drop out unexpectedly, and so we will work towards reducing this figure in the future.

In this paper, we are identifying user patterns in a group of people who have been shown to have a desire to perform physical exercise. Thus, predicting user behaviour will give us the capacity to identify and target users at risk of drop-out. As published by other authors, programme modifications and/or psychological interventions that specifically targeted at these subgroups will allow for behavioral modifications which can, eventually, lead to increased levels of physical activity on the part of these users [71]. Increased physical activity entails various physical, mental and psychological benefits and constitutes one of the best ways to prevent health problems [2]. Greater adherence to regular physical activity would most likely have a very significant impact on public health and this fact needs to be acknowledged. Further steps are required, until the tools required to ensure effective motivation are found that may help our communities be more physically active.

Our work was developed via a general approach, so as to take that it takes advantage of the models already trained to predict the workouts, instead of training the model for each user who joins the application. In this way, the processing time for the system is and will remain low, even when the number of users becomes high. This approach highlights the characteristics of flexibility and resource efficiency, which are within the definition of “Industry 4.0” and its two directions in terms of development [72]. In order to advance to the industrialization stage and contribute to the development of 4.0 applications, our proposed system could next be deployed in production.

Future processes could study more flexible longitudinal periods (other than 4 months), while variables of both engagement to the exercise plan and user motivation (intrinsic vs. extrinsic) could be included, possibly by previously applying a motivational questionnaire. This would enable analysts to more accurately identify user patterns and predict training behaviour. Fitness app developers would, accordingly, be in a position to undertake motivational intervention to promote training adherence and reduce app attrition.

## 5. Conclusions

Our paper adds to the scarcely researched area of training behaviour in fitness app users. There is still no consensus as to the exact definition of fitness app adherence, and there would seem not to be any previous research work that uses a deep learning approach to predict fitness app adherence over time. To the best of our knowledge, this is the first framework whose aim is to predict user adherence to training via a fitness app. The framework consists of two main stages: (i) characterization of users into user groups, based on their training behavior during the first three months; and (ii) the regression prediction for new users via an ensemble approach. Our results show that it is possible to take advantage of stored time-dependent data, in order to predict adherence over a given period of time. From the features studied, training frequency seems to be more relevant than time spent in training. For their part, our ensembles consisted of DL regressors and reflected good performance metrics. In the near future, we plan to incorporate demographic factors, as well as involvement variables in the workout regimen and user motivation, (intrinsic vs. extrinsic) into the DL architectural design. Additionally, we believe flexible longitudinal periods would be worth studying, the stages for which, we expect to be using a larger user database, which should enable thorough testing. The approach should focus on adhering to the principles of flexibility and resource efficiency, which will be essential in the creation of industrial 4.0 applications.

## Figures and Tables

**Table 1:** Data acquired using MH App.

**Personal Data**	
Age	Date of Birth
Gender	Male or Female
city	City and country
**Fitness data**	
Weight	Kg
Height	Cm
Body fat	Individual body fat estimation (%)
Body type	Thin, normal or overweight
**User goals**	
Body fat goal	Desired body fat goal (%)
User goal	Lose weight Stay healthy Gain strength
**Information application data**	
Profile creation	Date
App downloads	Date/s and number of downloads
App visits	Number of visits (total, per day, per week, per month)
Training plan purchased	Plan type
Training programme used	Programme type
Total sessions performed	Number of sessions Degree of difficulty of the sessions
Total sessions completed	Number of sessions
Completed sessions	Type of the most completed session Type of the most discarded session Number of sessions per week Date and hour of session completion Duration Finished YES/NO

**Table 2:** Characteristics of the participants who met the inclusion criteria.

Features	Age	Weight (Kgs)	Height (cm)	BMI	Body Fat	Objective of Body Fat
**Mean**	40	71.4	170.6	24.5	24.1	13.0
**Standard Deviation**	8	13.7	9.3	4.0	8.5	3.5
**min**	19	37.0	150.0	16.2	6.0	5.0
**25%**	34	62.0	164.0	22.0	20.0	10.0
**50%**	41	70.0	170.0	23.8	25.0	15.0
**75%**	47	79.9	177.5	25.9	30.0	15.0
**Max**	66	129.0	201.0	50.4	60.0	25.0

**Table 3:** Descriptive data for monthly training sessions.

Features	First Month	Second Month	Third Month	Fourth Month
**Mean [s]**	398.95	393.74	377.83	318.73
**Standard Deviation [s]**	594.89	632.84	631.76	620.22
**min [s]**	0.00	0.00	0.00	0.00
**25%**	22.03	0.00	0.00	0.00
**50%**	161.38	60.45	14.23	0.00
**75%**	514.50	604.14	553.71	466.95
**Max [s]**	4106.47	3643.43	3522.57	4524.93

**Table 4:** Frequency of users in ranges of exercise time per day.

Features	Number of Users First Month	Number of Users Second Month	Number of Users Third Month	Number of Users Fourth Month
**Time = 0 s**	41	106	121	132
**Time = [0 s–300 s]**	102	49	40	45
**Time = [300 s–1800 s]**	96	81	74	61
**Time = [1800 s–3600 s]**	4	9	11	6
**Time = [3600 s–7200 s]**	3	1	0	2

**Table 5:** Proposed framework algorithm: training.

1. Prepare the data: pre-processing, cleaning, scaling, splitting
2. Train clustering algorithms with all users with workout sessions over first three months
3. **For each** user **in** all users:
3.1. Configure architecture and set parameters to tune
3.2. **For each** parameters combination **in** the grid:
3.2.1. Train regression algorithms (LSTM, SVR)
3.2.2. Select the best model based on Mean absolute error (MAE)
3.2.3. Save the best model
4. Build ensemble of clusters with the best regression algorithms for each user, and with the corresponding clusters built in step 2

**Table 6:** Proposed framework algorithm: testing new users.

1. Prepare new user data: pre-processing, cleaning, scaling, splitting
2. Obtain user features from workouts sessions of first three months
3. Select the corresponding ensemble of models according to clustering result
4. **For each** model **in** ensemble:
4.1. Predict data of fourth month with the trained models
4.2. Save prediction
5. Obtain the result of workout in fourth month by calculating the mean of all predictions
6. Apply rule to determine adherence
7. Classify user adherence to MH fitness app

**Table 7:** Summarised input data.

	Value	Description
Total users	246	Total number of users, with at least 4 months of data
Longitudinal Period selected	120 [days]	Longitudinal period of training sessions based on state of the art
Days selected for training	90 [days]	Days selected to train the artificial intelligence system
Days selected for testing	30 [days]	Days selected to test the artificial intelligence system
Scores	Sessions [s]	Array with training session data for users over 4 months
Output class 1	112	Users who are adherent during the fourth month
Output class 0	134	Users who are not adherent during the fourth month

**Table 8:** Basic k-means algorithm.

1. Select K points as initial centroids
2. **while**
2.1. K clusters by assigning each point to its closest centroid
2.2. Recompute the centroid of each cluster
3. **until** centroids do not change

**Table 9:** Basic BIRCH algorithm.

1. **For each** record $x_{i}$ **in** set of elements *D*:
1.1. Determine correct leaf node for $x_{i}$ insertion
1.2. **If** threshold condition is not violated then:
1.2.1. Add $x_{i}$ to cluster and update CF
1.3. **else if** threshold condition is violated:
1.3.1. Insert $x_{i}$ as single cluster and update CF
2. Apply an existing clustering algorithm to the sub-clusters, with a view to combining these sub-clusters into clusters

**Table 10:** Basic Affinity propagation algorithm.

1. Set “availabilities” to zero i.e., ∀ i,k: a(i,k) = 0
1.2. **While** responsibility and availability matrices are updated until they converge:
1.3. Calculate similarity matrix
1.4. Calculate responsibility matrix
1.5. Calculate availability matrix
2. Cluster assignments corresponding to the highest criterion values of each row is designated as the exemplar i.e., argmax_k [a(i,k) + r(i,k)]

**Table 11:** Description of features used for clustering.

Variable	Description
mean_first_month	Mean of completed sessions in the first month of training (seconds)
mean_second_month	Mean of completed sessions in the second month of training (seconds)
mean_third_month	Mean of completed sessions in the three month of training (seconds)
missed_first_month	The number of skipped sessions in the first month
missed_second_month	The number of skipped sessions in the second month
missed_third_month	The number of skipped sessions in the third month
mean_week_1	Mean of completed sessions in the first month of training (seconds)
mean_week_2	Mean of completed sessions in the first week of training (seconds)
mean_week_3	Mean of completed sessions in the second week of training (seconds)
...	...
mean_week_12	Mean of completed sessions in the 12th month of training (seconds)

**Table 12:** Hyperparameters used in the LSTM ensemble models.

Hyperparameters	Values
lookback	7
Number of epochs	50
Number of hidden layers	4
Number of LSTM layers	1
Number of dense layers	3
Activation function	relu
Optimizer	adam
Loss	mse
Early stopping	Patience:15 Monitor: loss
**Dropout**	**[ 0.2, 0.4, 0.6]**
**Batch size**	**[1, 2, 4]**
**Number of Neurons**	**[50, 75, 100, 125, 150]**

**Table 13:** Metrics for Ensemble without clusters.

Accuracy	Recall	Precision	Specificity	F1_score
0.7276	0.4196	0.9592	0.9851	0.5839

**Table 14:** Metrics, applying different configurations for k-means clustering—SVR.

		Features Selected			
**Parameters**	**Metrics**	mean_fm, mean_sm, mean_tm	**missed_first_month,** **missed_second_month,** **missed_third_month**	week_0, week_1, week_2, ..., week_11	missed_first_month, missed_second_month, missed_third_month, mean_fm, mean_sm, mean_tm, week8, week9 week10, week 11
**k = 3**	Accuracy	0.7073	**0.7764**	0.7398	0.7195
	Recall	0.375	**0.5268**	0.4464	0.4018
	Precision	0.9545	**0.9672**	0.9615	0.9574
	Specificity	0.9851	**0.9851**	0.9851	0.9851
	F1_score	0.5385	**0.6821**	0.6098	0.566
k = 4	Accuracy	0.7073	0.7358	0.7398	0.7236
	Recall	0.3661	0.4643	0.4375	0.4107
	Precision	0.9762	0.9123	0.98	0.9583
	Specificity	0.9925	0.9627	0.9925	0.9851
	F1_score	0.5325	0.6154	0.6049	0.575

**Table 15:** Metrics, applying different configurations for BIRCH clustering—SVR.

		Features Selected			
**Parameters**	**Metrics**	mean_fm, mean_sm, mean_tm	**missed_first_month,** **missed_second_month,** **missed_third_month**	week_0, week_1, week_2, ..., week_11	missed_first_month, missed_second_month, missed_third_month, mean_fm, mean_sm, mean_tm, week8, week9 week10, week 11
k = 4, threshold = 0.001	Accuracy	0.687	0.7764	0.7764	0.7236
	Recall	0.3214	0.5268	0.5268	0.4018
	Precision	0.973	0.9672	0.9672	0.9783
	Specificity	0.9925	0.9851	0.9851	0.9925
	F1_score	0.4832	0.6821	0.6821	0.5696
**k = 4,** **threshold = 0.1**	Accuracy	0.687	**0.7764**	0.752	0.7154
	Recall	0.3214	**0.5268**	0.4732	0.3929
	Precision	0.973	**0.9672**	0.9636	0.9565
	Specificity	0.9925	**0.9851**	0.9851	0.9851
	F1_score	0.4832	**0.6821**	0.6347	0.557
k = 4, threshold = 100	Accuracy	0.7602	0.752	0.7114	0.752
	Recall	0.4911	0.4732	0.375	0.4732
	Precision	0.9649	0.9636	0.9767	0.9636
	Specificity	0.9851	0.9851	0.9925	0.9851
	F1_score	0.6509	0.6347	0.5419	0.6347

**Table 16:** Metrics, applying different configurations for Affinity propagation clustering—SVR.

		Parameters: Damping							
Features selected	Metrics	**0.66**	0.68	0.7	0.72	0.74	0.76	0.78	0.8
**mean_fm,** **mean_sm,** **mean_tm,**	Accuracy	**0.752**	0.7114	0.7114	0.7154	0.7154	0.7195	0.7154	0.7195
	Recall	**0.4732**	0.375	0.375	0.3839	0.3839	0.3929	0.3839	0.3929
	Precision	**0.9636**	0.9767	0.9767	0.9773	0.9773	0.9778	0.9773	0.9778
	Specificity	**0.9851**	0.9925	0.9925	0.9925	0.9925	0.9552	0.9925	0.9925
	F1_score	**0.6347**	0.5419	0.5419	0.5513	0.5513	0.5605	0.5513	0.5605
missed_first_month, missed_second_month, missed_third_month	Accuracy	0.6992	0.6911	0.687	0.687	0.687	0.687	0.687	0.687
	Recall	0.3393	0.3214	0.3125	0.3125	0.3125	0.3125	0.3125	0.3125
	Precision	1.0	1.0	1.0	1.0	1.0	1.0	1.0	1.0
	Specificity	1.0	1.0	1.0	1.0	1.0	1.0	1.0	1.0
	F1_score	0.5067	0.4865	0.4762	0.4762	0.4762	0.4762	0.4762	0.4762
week_0, week_1 week_2,...,week_11	Accuracy	0.752	0.7317	0.7317	0.7317	0.7358	0.752	0.7317	0.7317
	Recall	0.4732	0.4196	0.4196	0.4196	0.4196	0.4732	0.4196	0.4196
	Precision	0.9636	0.9792	0.9792	0.9792	1.0	0.9636	0.9792	0.9792
	Specificity	0.9851	0.9925	0.9925	0.9925	1.0	0.9851	0.9925	0.9925
	F1_score	0.6347	0.5875	0.5875	0.5875	0.5912	0.6347	0.5875	0.5875
**missed_first_month**, **missed_second_month**, **missed_third_month**, **mean_fm**, **mean_sm**, **mean_tm**, **week8**, **week9**, **week10**, **week 11**	Accuracy	**0.752**	0.7317	0.7317	0.7317	0.7358	0.7317	0.7317	0.7317
	Recall	**0.4732**	0.4107	0.4107	0.4107	0.4196	0.4107	0.4107	0.4107
	Precision	**0.9636**	1.0	1.0	1.0	1.0	1.0	1.0	1.0
	Specificity	**0.9851**	1.0	1.0	1.0	1.0	1.0	1.0	1.0
	F1_score	**0.6347**	0.5823	0.5823	0.5823	0.5912	0.5823	0.5823	0.5823
Features selected	Metrics	0.82	0.84	0.86	0.88	0.9	0.92	0.94	0.96
mean_fm, mean_sm, mean_tm,	Accuracy	0.7033	0.7033	0.7033	0.7033	0.7398	0.7398	0.7398	0.7398
	Recall	0.3571	0.3571	0.3571	0.3571	0.4464	0.4464	0.4464	0.4464
	Precision	0.9756	0.9756	0.9756	0.9756	0.9615	0.9615	0.9615	0.9615
	Specificity	0.9925	0.9925	0.9925	0.9925	0.9851	0.9851	0.9851	0.9851
	F1_score	0.5229	0.5229	0.5229	0.5229	0.6098	0.6098	0.6098	0.6098
missed_first_month, missed_second_month, missed_third_month	Accuracy	0.6911	0.687	0.687	0.6911	0.6911	0.6911	0.6992	0.7195
	Recall	0.3214	0.3125	0.3125	0.3214	0.3214	0.3214	0.3393	0.3929
	Precision	1.0	1.0	1.0	1.0	1.0	1.0	1.0	0.9778
	Specificity	1.0	1.0	1.0	1.0	1.0	1.0	1.0	0.9925
	F1_score	0.4865	0.4762	0.4762	0.4865	0.4865	0.4865	0.5067	0.5605
week_0, week_1 week_2,...,week_11	Accuracy	0.7073	0.7317	0.5875	0.7073	0.7073	0.7317	0.7317	0.7195
	Recall	0.3661	0.4196	0.4196	0.3661	0.3661	0.4196	0.4196	0.4018
	Precision	0.9762	0.9792	0.9792	0.9762	0.9762	0.9792	0.9792	0.9574
	Specificity	0.9925	0.9925	0.9925	0.9925	0.9925	0.9925	0.9925	0.9851
	F1_score	0.5325	0.5875	0.5875	0.5325	0.5325	0.5875	0.5875	0.566
missed_first_month, missed_second_month, missed_third_month, mean_fm, mean_sm, mean_tm, week8, week9, week10, week 11	Accuracy	0.7317	0.7317	0.7317	0.7195	0.752	0.752	0.6951	0.6992
	Recall	0.4107	0.4107	0.4107	0.3839	0.4732	0.4732	0.3393	0.3482
	Precision	1.0	1.0	1.0	1.0	0.9636	0.9636	0.9744	0.975
	Specificity	1.0	1.0	1.0	1.0	0.9851	0.9851	0.9925	0.9925
	F1_score	0.5823	0.5823	0.5823	0.5548	0.6347	0.6347	0.5033	0.5132

**Table 17:** Metrics, applying different configurations for k-means clustering—LSTM.

		Features Selected			
**Parameters**	**Metrics**	mean_fm, mean_sm, mean_tm,	**missed_first_month,** **missed_second_month,** **missed_third_month**	week_0, week_1 week_2,...,week_11	missed_first_month, missed_second_month, missed_third_month, mean_fm, mean_sm, mean_tm, week8, week9 week10, week 11
**k = 3**	Accuracy	0.7967	**0.8496**	0.7805	0.7967
	Recall	0.5714	**0.7054**	0.5268	0.5625
	Precision	0.9697	**0.9518**	0.9833	0.9844
	Specificity	0.9851	**0.9701**	0.9925	0.9925
	F1_score	0.7191	**0.8103**	0.686	0.7159
k = 4	Accuracy	0.8089	0.7967	0.7764	0.8089
	Recall	0.5714	0.6161	0.5179	0.5982
	Precision	0.9697	0.9452	0.9831	0.971
	Specificity	0.9851	0.9701	0.9925	0.9851
	F1_score	0.7191	0.7459	0.6784	0.7403

**Table 18:** Metrics, applying different configurations for BIRCH clustering—LSTM.

		**Features Selected**				
**Parameters**	Metrics	mean_fm, mean_sm, mean_tm,	missed_first_month, missed_second_month, missed_third_month	week_0, week_1 week_2,...,week_11	**missed_first_month,** **missed_second_month,** **missed_third_month,** **mean_fm, mean_sm,** **mean_tm, week8, week9,** **week10, week 11**
k = 4, threshold = 0.001	Accuracy	0.8089	0.7967	0.7967	0.8333
	Recall	0.5893	0.5625	0.5893	0.6518
	Precision	0.9851	0.9844	0.9429	0.9733
	Specificity	0.9925	0.9925	0.9701	0.9851
	F1_score	0.7374	0.7159	0.7253	0.7807
**k = 4,** **threshold = 0.1**	Accuracy	0.8089	0.7967	0.7967	**0.8415**
	Recall	0.5893	0.5625	0.5893	**0.6607**
	Precision	0.9851	0.9844	0.9429	**0.9867**
	Specificity	0.9925	0.9925	0.9701	**0.9925**
	F1_score	0.7374	0.7159	0.7253	**0.7914**
k = 4, threshold = 100	Accuracy	0.7398	0.7276	0.7805	0.7276
	Recall	0.4286	0.4196	0.5536	0.4196
	Precision	1.0	0.9592	0.9394	0.9592
	Specificity	1.0	0.9851	0.9701	0.9851
	F1_score	0.6	0.5839	0.6966	0.5839

**Table 19:** Metrics, applying different configurations for Affinity propagation clustering—LSTM.

		Parameters: Damping							
Features selected	Metrics	0.66	**0.68**	0.7	0.72	0.74	0.76	0.78	0.8
mean_fm, mean_sm, mean_tm,	Accuracy	0.7276	0.8293	0.8293	0.8252	0.8252	0.8252	0.8252	0.8089
	Recall	0.4196	0.6786	0.6786	0.6696	0.6696	0.6696	0.6696	0.5893
	Precision	0.9592	0.9268	0.9268	0.9259	0.9259	0.9259	0.9259	0.9851
	Specificity	0.9851	0.9552	0.9552	0.9552	0.9552	0.9552	0.9552	0.9925
	F1_score	0.5839	0.7835	0.7835	0.7772	0.7772	0.7772	0.7772	0.7374
missed_first_month, missed_second_month, missed_third_month	Accuracy	0.8496	0.8496	0.7967	0.7967	0.7967	0.7967	0.7967	0.7967
	Recall	0.6875	0.6964	0.5893	0.5893	0.5893	0.5893	0.5893	0.5893
	Precision	0.9747	0.963	0.9429	0.9429	0.9429	0.9429	0.9429	0.9429
	Specificity	0.9851	0.9776	0.9701	0.9701	0.9701	0.9701	0.9701	0.9701
	F1_score	0.8063	0.8083	0.7253	0.7253	0.7253	0.7253	0.7253	0.7253
week_0, week_1 week_2,...,week_11	Accuracy	0.8577	0.8577	0.8577	0.8577	0.8618	0.8577	0.8577	0.8577
	Recall	0.7411	0.7411	0.7411	0.7411	0.75	0.7411	0.7411	0.7411
	Precision	0.9326	0.9326	0.9326	0.9326	0.9333	0.9326	0.9326	0.9326
	Specificity	0.9552	0.9552	0.9552	0.9552	0.9552	0.9552	0.9552	0.9552
	F1_score	0.8259	0.8259	0.8259	0.8259	0.8317	0.8259	0.8259	0.8259
**missed_first_month,** **missed_second_month,** **missed_third_month,** **mean_fm, mean_sm,** **mean_tm, week8, week9,** **week10, week 11**	Accuracy	0.7276	**0.8775**	0.8333	0.8333	0.8333	0.8333	0.8333	0.8333
	Recall	0.4196	**0.7748**	0.6964	0.6964	0.6964	0.6964	0.6964	0.6964
	Precision	0.9592	**0.9451**	0.9176	0.9176	0.9176	0.9176	0.9176	0.9176
	Specificity	0.9851	**0.9627**	0.9478	0.9478	0.9478	0.9478	0.9478	0.9478
	F1_score	0.5839	**0.8514**	0.7919	0.7919	0.7919	0.7919	0.7919	0.7919
Features selected	Metrics	0.82	0.84	0.86	0.88	0.9	**0.92**	0.94	0.96
mean_fm, mean_sm, mean_tm,	Accuracy	0.8252	0.8252	0.8252	0.8252	0.7561	0.7561	0.7561	0.7561
	Recall	0.6696	0.6696	0.6696	0.6696	0.4732	0.4732	0.4732	0.4732
	Precision	0.9259	0.9259	0.9259	0.9259	0.9815	0.9815	0.9815	0.9815
	Specificity	0.9552	0.9552	0.9552	0.9552	0.9925	0.9925	0.9925	0.9925
	F1_score	0.7772	0.7772	0.7772	0.7772	0.6386	0.6386	0.6386	0.6386
missed_first_month, missed_second_month, missed_third_month	Accuracy	0.7967	0.7967	0.7967	0.7967	0.7967	0.8577	0.813	0.813
	Recall	0.5893	0.5893	0.5893	0.5893	0.5893	0.7143	0.6161	0.6161
	Precision	0.9429	0.9429	0.9429	0.9429	0.9429	0.9639	0.9583	0.9583
	Specificity	0.9701	0.9701	0.9701	0.9701	0.9701	0.9776	0.9776	0.9776
	F1_score	0.7253	0.7253	0.7253	0.7253	0.7253	0.8205	0.75	0.75
week_0, week_1 week_2,...,week_11	Accuracy	0.8577	0.8618	0.8618	0.8577	0.8577	0.8659	0.8659	0.8496
	Recall	0.7411	0.7411	0.7411	0.7411	0.7411	0.7411	0.7411	0.6786
	Precision	0.9326	0.9432	0.9432	0.9326	0.9326	0.954	0.954	0.987
	Specificity	0.9552	0.9627	0.9627	0.9552	0.9552	0.9701	0.9701	0.9925
	F1_score	0.8259	0.83	0.83	0.8259	0.8259	0.8342	0.8342	0.8042
missed_first_month, missed_second_month, missed_third_month, mean_fm, mean_sm, mean_tm, week8, week9, week10, week 11	Accuracy	0.8333	0.8333	0.8374	0.8333	0.7276	0.7276	0.7927	0.7886
	Recall	0.6964	0.6964	0.6607	0.6964	0.4196	0.4196	0.5536	0.5446
	Precision	0.9176	0.9176	0.9737	0.9176	0.9592	0.9592	0.9841	0.9839
	Specificity	0.9478	0.9478	0.9851	0.9478	0.9851	0.9851	0.9925	0.9925
	F1_score	0.7919	0.7919	0.7872	0.7919	0.5839	0.5839	0.7086	0.7011

**Table 20:** Confusion Matrix: 4—Means.

Confusion Matrix	Predicted: No	Predicted: Yes
Actual: No	TN: 129	FP: 5
Actual: Yes	FN: 25	TP: 86

## Data Availability

Data are contained within the article.

## Abbreviations

The following abbreviations are used in this manuscript:

AP	Affinity Propagation
BIRCH	Balanced Iterative Reducing and Clustering using Hierarchies
CF	Cluster Feature
DL	Deep Learning
FN	False negatives
FP	False positives
LSTM	Long Short Term Memory
MAE	Mean Absolute Error
MH	Mammoth Hunters
ML	Machine learning
PA	Physical activity
RNN	Recurrent Neural Networks
SSE	Sum of Squared Errors
SVM	Support vector machine
SVR	Support vector regression
TN	True negatives
TP	True positives
WHO	World Health Organization
